# Development and validation of a risk prediction model for activities of daily living dysfunction in stroke survivors

**DOI:** 10.3389/fneur.2025.1529724

**Published:** 2025-05-30

**Authors:** Fangbo Lin, Nan Liu

**Affiliations:** Neurology Department, Fujian Medical University Union Hospital, Fuzhou, China

**Keywords:** stroke survivors, ADL dysfunction, prediction model, LASSO regression, nomogram

## Abstract

**Objective:**

Stroke is a leading cause of disability worldwide, imposing a significant burden on patients, families, and society. To create and verify a prediction model for activities of daily living (ADL) dysfunction in stroke survivors, pinpoint key predictors, and analyze the traits of those at risk.

**Methods:**

Data from the China Health and Retirement Longitudinal Study wave 5 was used in this cross-sectional study. 1,131 stroke survivors were included and split into training and testing sets. The least absolute shrinkage and selection operator regression and multivariate logistic regression were applied for model development. Model performance was evaluated using the area under the receiver operating characteristic curve(AUC), calibration plots, and decision curve analysis. SHapley Additive exPlanations values were calculated to understand predictor importance.

**Results:**

Six variables (age, the 10-item Center for Epidemiologic Studies Depression Scale score, memory disorder, self-rated health, pain count, and heavy physical activity) were identified as significant predictors. The model showed good discriminatory power (training set AUC = 0.804, testing set AUC = 0.779), accurate calibration, and clinical utility.

**Conclusion:**

A prediction model for ADL dysfunction in stroke survivors was successfully developed and validated. It can help in formulating personalized medical plans, potentially enhancing stroke survivors' ADL ability and quality of life.

## 1 Introduction

Stroke is a leading cause of disability worldwide, imposing a significant burden on patients, families, and society (1). Despite advancements in acute phase stroke treatment, a large number of stroke survivors experience limitations in activities of daily living (ADL) (2), which severely impacts their quality of life. Understanding the factors associated with ADL dysfunction in stroke survivors and predicting its occurrence at an early stage is crucial for developing targeted interventions.

Previous studies have investigated various factors related to post-stroke ADL dysfunction (3, 4), but there is still a lack of a comprehensive and accurate prediction model. Identifying individuals at high risk of ADL dysfunction early can enable timely implementation of rehabilitation strategies and management plans, potentially improving their functional outcomes and quality of life (5, 6).

In this study, we aimed to develop and validate a prediction model for ADL dysfunction in stroke survivors. By analyzing data from a large scale dataset, we aimed to identify key predictors, understand the characteristics of stroke survivors at risk of ADL dysfunction, and provide a basis for personalized medical care and improved management of this patient population.

## 2 Methods

### 2.1 Study design

This study adopted a cross-sectional design, which is suited for exploring associations between risk factors and functional outcomes in stroke survivors. Cross-sectional studies are advantageous for identifying potential predictors of post-stroke disability, providing a basis for future longitudinal investigations. Data were obtained from wave 5 of the China Health and Retirement Longitudinal Study (CHARLS). The dataset is publicly accessible via the official CHARLS website (http://CHARLS.pku.edu.cn). The study adhered to ethical norms and received approval from the Biomedical Ethics Committee of Peking University, China (IRB00001052-11,015) (7). All procedures followed the principles outlined in the Declaration of Helsinki, and informed consent was obtained from all participants. In this study, patients and the public were not involved in the design, conduct, reporting, or dissemination plans of the research. This study adhered to the Transparent Reporting of a multivariable prediction model for Individual Prognosis or Diagnosis (TRIPOD) (8).

### 2.2 Study population

In the CHARLS database, stroke status was determined through self-reported responses to the question: “Have you ever been diagnosed with a stroke by a doctor?” This self-reported approach may lead to potential misclassification, which was considered in the study's limitations. ADL in two main categories: Basic ADL (BADL), which includes six essential tasks: dressing, bathing, eating, transferring (getting in and out of bed), toileting, and controlling urination and defecation. Instrumental ADL (IADL), which involves more complex activities, such as managing housework, cooking, shopping, financial management, and medication adherence. We used a questionnaire-based survey method to assess ADL dysfunction. If a stroke survivor was unable to independently complete any of the tasks listed under BADL or IADL, they were classified as having ADL dysfunction. Inclusion criteria: History of stroke; Ability to cooperate in completing the ADL screening. Exclusion criteria: No history of stroke or uncertain stroke diagnosis; Missing key variables; Pre-existing ADL dysfunction before stroke. Initially, 1,381 individuals with a self-reported history of stroke were identified. After excluding individuals with more than 30% missing data, the final analysis included 1,131 stroke survivors.

### 2.3 Candidate predictor variables

Predictor selection was based on prior literature and clinical expertise (9–11). Although stroke characteristics (e.g., lesion location, infarct size) influence prognosis, these variables were not recorded in CHARLS. Instead, we examined demographic, behavioral, health, and socioeconomic factors available in the dataset. Basic factors: age, gender, residence (urban/rural), education level, marital status, and life satisfaction (five-point Likert scale). Behavioral factors: sleep duration, smoking, alcohol consumption, social activity participation (eight categories), and physical activity levels (light, moderate, heavy). Total energy expenditure from physical activity was calculated using metabolic equivalent (MET) scores. Health status and medical conditions: self-rated health (five levels), hypertension, diabetes, cancer, cardiac disease, mental disorders, and the 10-item Center for Epidemiologic Studies Depression Scale (CESD-10). Family and economic factors: household size, financial support from children/parents, and number of surviving children.

### 2.4 Statistical analysis

All statistical analyses were conducted using R software. Continuous variables were reported as medians and interquartile ranges, while categorical variables were presented as proportions. Between-group comparisons were performed using the Wilcoxon rank-sum test for continuous data and the Chi-square test or Fisher's exact test for categorical data. To develop the ADL dysfunction prediction model, the dataset was randomly split (6:4) into a training set (*n* = 678) and a testing set (*n* = 453). We applied the least absolute shrinkage and selection operator (LASSO) regression to identify key predictors while addressing multicollinearity. Optimal tuning parameters (λ) were selected via ten-fold cross-validation. Selected variables were incorporated into a multivariate logistic regression model, with predictors retained at *P* < 0.05. Model performance was assessed using the area under the receiver operating characteristic (ROC) curve (AUC) for discrimination, calibration plots for agreement between predicted and observed outcomes, and decision curve analysis (DCA) for clinical utility. SHapley Additive exPlanations (SHAP) values were computed to interpret predictor importance.

## 3 Results

### 3.1 Flow chart

**Figure 1:**
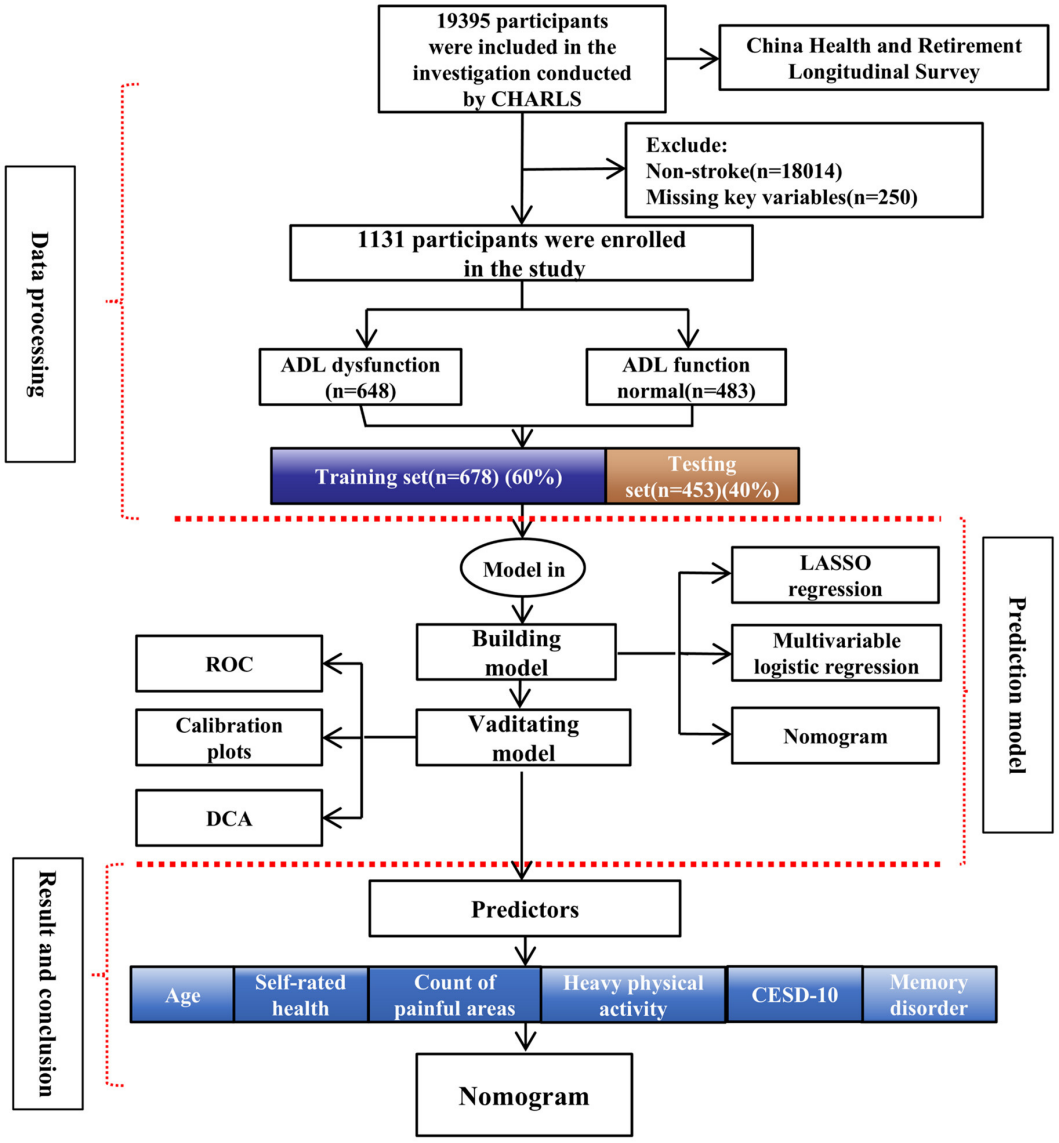
Flow chart of the study.

The study flow chart is presented in Figure 1.

### 3.2 Baseline characteristics

A total of 1,131 stroke survivors were included in this study. The demographic and clinical characteristics of the participants are summarized in Table 1. The cohort consisted of 473 male participants (41.8%) and 658 female participants (58.2%), with an average age of 67 years. Among these stroke survivors, 57.3% experienced difficulties with ADL. Several factors showed significant differences (*p* < 0.05) between stroke survivors with normal and impaired ADL function, including age, gender, education level, life satisfaction, CESD-10 score, and health status factors (e.g., hypertension, lung disease, arthritis). Additionally, factors such as social activity, sleep duration, pain count, and physical activity levels were also significantly associated with ADL dysfunction.

**Table 1:** Participant characteristics.

**Subject**	**ADL function normal**	**ADL dysfunction**	***p* value**
*N*	483	648	
Family size	2.00 (2.00–3.00)	2.00 (2.00–3.00)	0.487
Health child	2.00 (2.00–3.00)	3.00 (2.00–4.00)	0.028
Children's economic support	2500.00 (500.00–6500.00)	2515.00 (687.50–7000.00)	0.229
Parent's economic support	100.00 (0.00–2000.00)	100.00 (0.00–1200.00)	0.667
CESD-10	7.00 (3.50–12.00)	14.00 (9.00–19.00)	<0.001
Life satisfaction	3.00 (3.00–4.00)	3.00 (3.00–4.00)	<0.001
Count of painful areas	1.00 (0.00–4.00)	3.00 (1.00–7.25)	<0.001
Sleep duration	6.00 (5.00–7.50)	5.00 (4.00–7.00)	<0.001
Total categories of social activities	1.00 (0.00–1.00)	0.00 (0.00–1.00)	<0.001
Age	67.00 (60.00-71.00)	69.00 (62.75-74.00)	<0.001
Education level	2.00 (1.00–3.00)	1.00 (1.00–3.00)	<0.001
Total metabolic output from physical activity	3814.00 (1485.00–7068.00)	1732.50 (462.00–4764.00)	<0.001
Episodic memory (0–10)	4.00 (3.00–5.50)	3.50 (2.00–5.00)	<0.001
Self-rated health	3.00 (2.00–3.00)	2.00 (1.00–3.00)	<0.001
**Gender**			
0	195 (40.37%)	363 (56.02%)	<0.001
1	288 (59.63%)	285 (43.98%)	
**Marry**			
0	91 (18.84%)	142 (21.91%)	0.206
1	392 (81.16%)	506 (78.09%)	
**Residence**			
0	200 (41.41%)	240 (37.04%)	0.136
1	283 (58.59%)	408 (62.96%)	
**Hip fracture**			
0	483 (100.00%)	630 (97.22%)	<0.001
1	0 (0.00%)	18 (2.78%)	
**Hypertension**			
0	166 (34.37%)	169 (26.08%)	0.003
1	317 (65.63%)	479 (73.92%)	
**Diabetes**			
0	360 (74.53%)	456 (70.37%)	0.122
1	123 (25.47%)	192 (29.63%)	
**Cancer**			
0	471 (97.52%)	629 (97.07%)	0.648
1	12 (2.48%)	19 (2.93%)	
**Lung disease**			
0	410 (84.89%)	491 (75.77%)	<0.001
1	73 (15.11%)	157 (24.23%)	
**Cardiac disease**			
0	318 (65.84%)	335 (51.70%)	<0.001
1	165 (34.16%)	313 (48.30%)	
**Mental disorder**			
0	461 (95.45%)	590 (91.05%)	0.004
1	22 (4.55%)	58 (8.95%)	
**Arthritis**			
0	251 (51.97%)	260 (40.12%)	<0.001
1	232 (48.03%)	388 (59.88%)	
**Dyslipidemia**			
0	261 (54.04%)	308 (47.53%)	0.030
1	222 (45.96%)	340 (52.47%)	
**Liver disease**			
0	434 (89.86%)	551 (85.03%)	0.017
1	49 (10.14%)	97 (14.97%)	
**Kidney disease**			
0	406 (84.06%)	485 (74.85%)	<0.001
1	77 (15.94%)	163 (25.15%)	
**Digestive disease**			
0	309 (63.98%)	382 (58.95%)	0.086
1	174 (36.02%)	266 (41.05%)	
**Asthma**			
0	452 (93.58%)	569 (87.81%)	0.001
1	31 (6.42%)	79 (12.19%)	
**Memory disorder**			
0	432 (89.44%)	506 (78.09%)	<0.001
1	51 (10.56%)	142 (21.91%)	
**Intense exercise**			
0	319 (66.05%)	506 (78.09%)	<0.001
1	164 (33.95%)	142 (21.91%)	
**Moderate exercise**			
0	233 (48.24%)	402 (62.04%)	<0.001
1	250 (51.76%)	246 (37.96%)	
**Light exercise**			
0	114 (23.60%)	156 (24.07%)	0.854
1	369 (76.40%)	492 (75.93%)	
**Alcohol consumption**			
0	318 (65.84%)	505 (77.93%)	<0.001
1	165 (34.16%)	143 (22.07%)	
**Smoking**			
0	239 (49.48%)	365 (56.33%)	0.022
1	244 (50.52%)	283 (43.67%)	

### 3.3 Prediction model development

**Figure 2:**
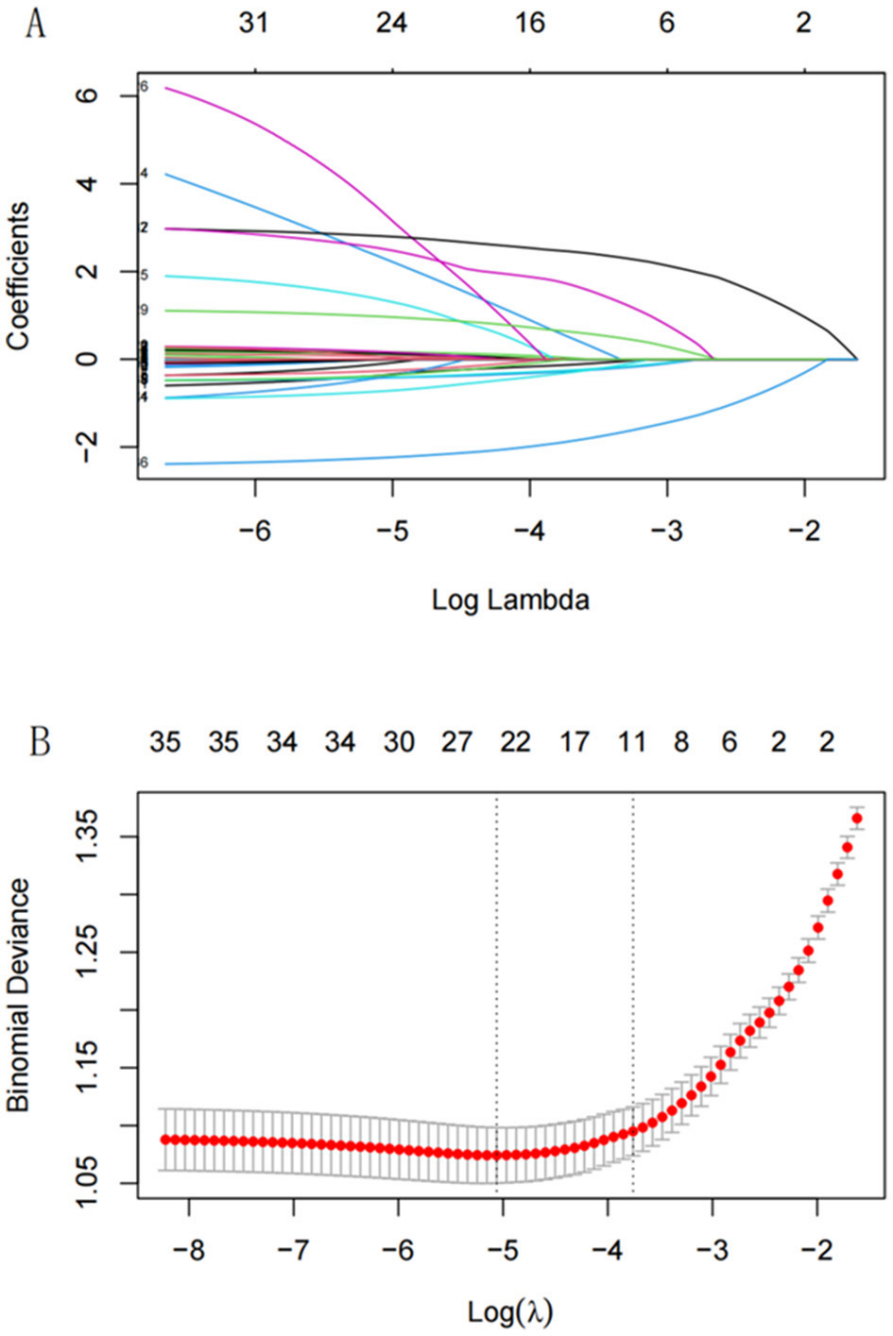
The LASSO plot.

**Figure 3:**
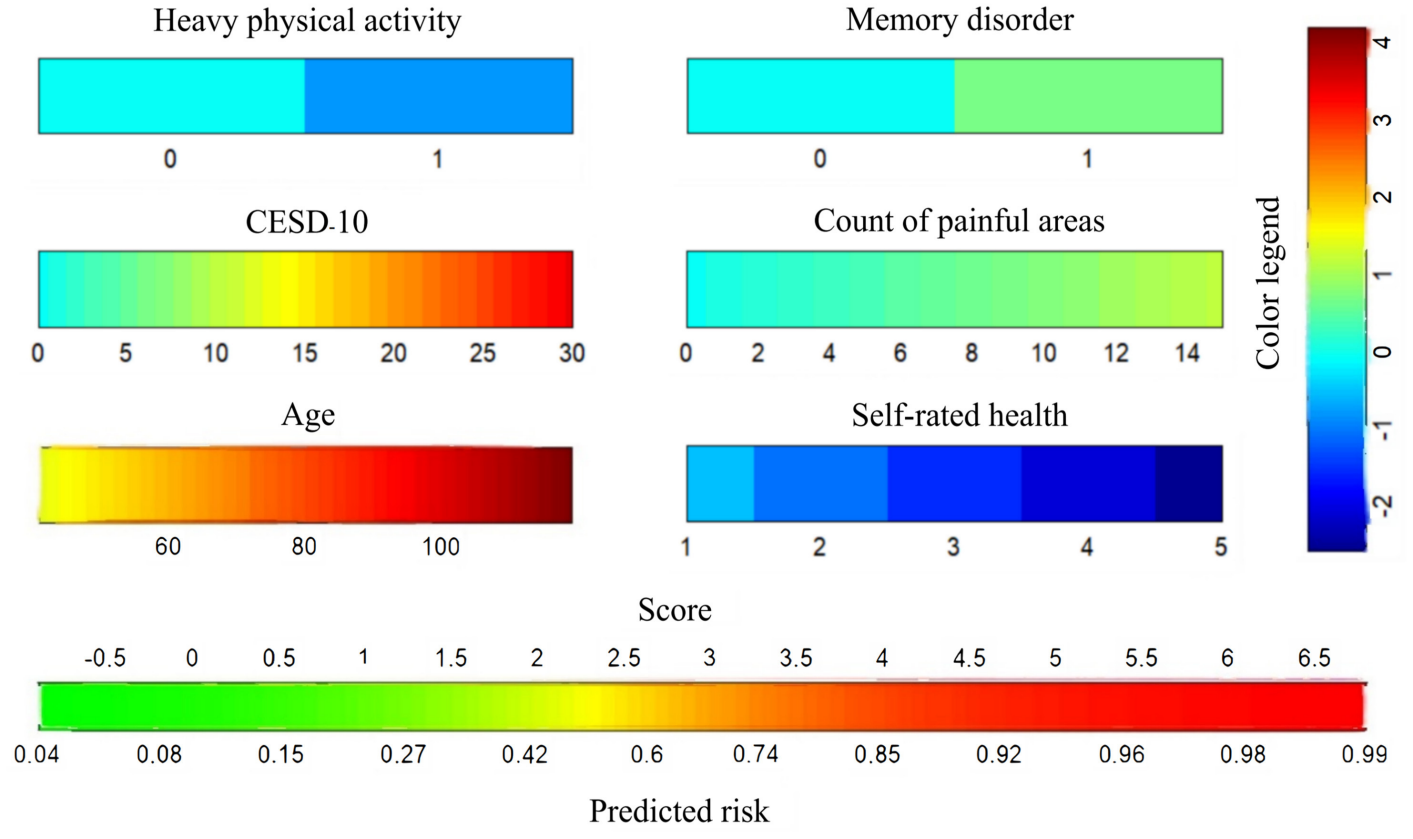
Nomogram to predict the probability of ADL dysfunction in stroke survivors.

LASSO regression was applied to identify the best predictors for ADL dysfunction, with predictors selected based on 10-fold cross-validation. The 11 significant variables identified included gender, age, hip fracture, CESD-10 score, memory disorder, pain areas, and levels of physical activity (Figure 2). These variables were then used in a multivariate logistic regression model, which selected the following significant predictors (*P* < 0.05): age, CESD-10 score, memory disorder, self-rated health, pain count, and heavy physical activity. The resulting predictive model was visualized through a nomogram, which allows for the quantitative assessment of ADL dysfunction risk in stroke survivors (Figure 3).

### 3.4 Prediction model validation

**Figure 4:**
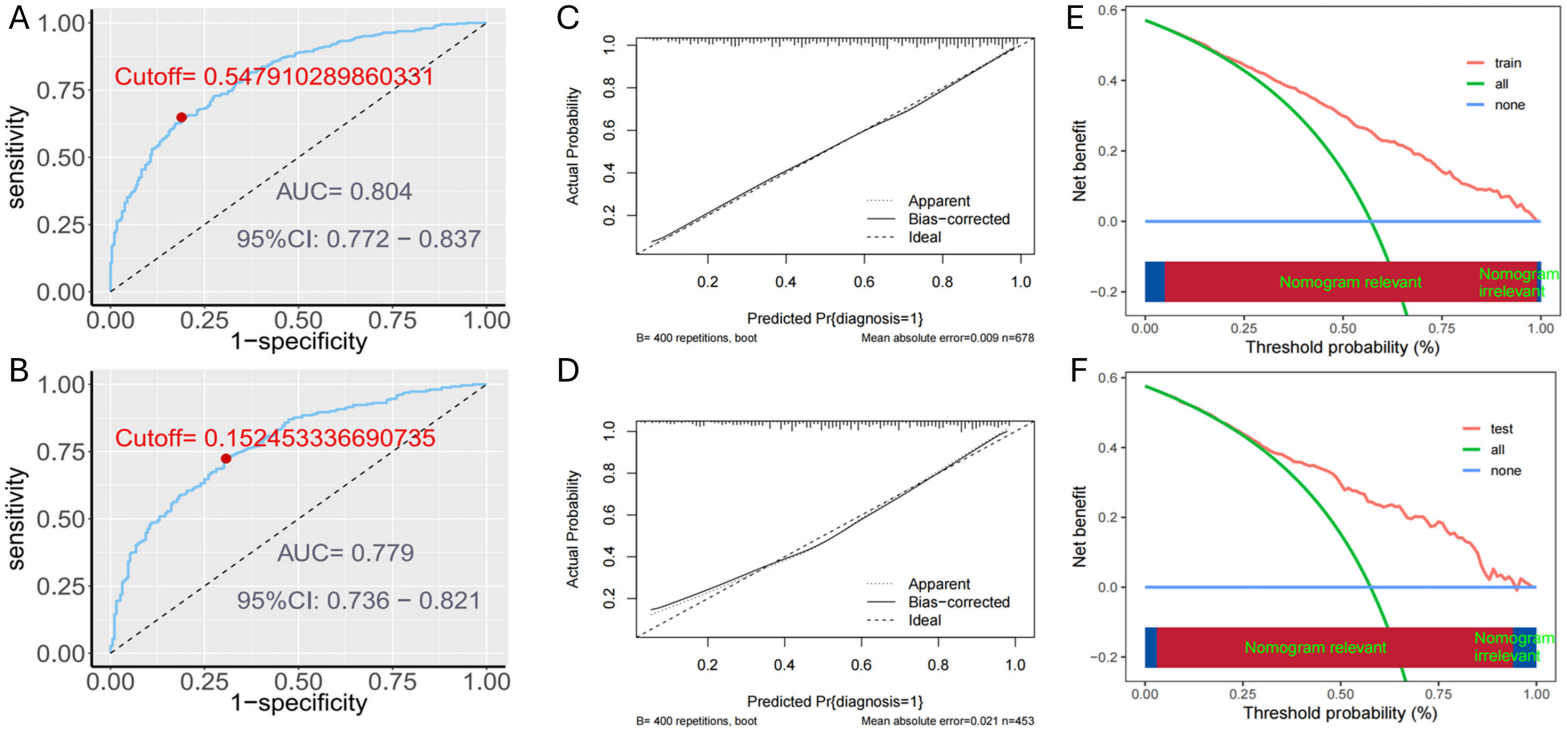
Assessment of the predictive accuracy of the nomogram: **(A)** ROC for the training set; **(B)** ROC for the testing set. Assessment of the predictive accuracy of the nomogram: **(C)** Calibration plot for the training set; **(D)** Calibration plot for the testing set. DCA curves of the nomogram: **(E)** The training set; **(F)** The testing set.

The predictive model's performance was assessed using the AUC. In the training set, the AUC value was 0.804 (95% CI: 0.772–0.837), and in the testing set, it was 0.779 (95% CI: 0.736–0.821), indicating good discriminatory power (Figures 4A, B). The nomogram's calibration curves (Figures 4C, D) showed alignment between predicted and observed probabilities of ADL dysfunction, confirming the model's accuracy and reliability. Clinical validity was assessed using DCA, shown in Figures 4E, F. The DCA demonstrated that the prediction model provided net benefits compared to the two extreme scenarios, suggesting its clinical utility in predicting ADL dysfunction.

### 3.5 Explanation of model characteristic variables

**Figure 5:**
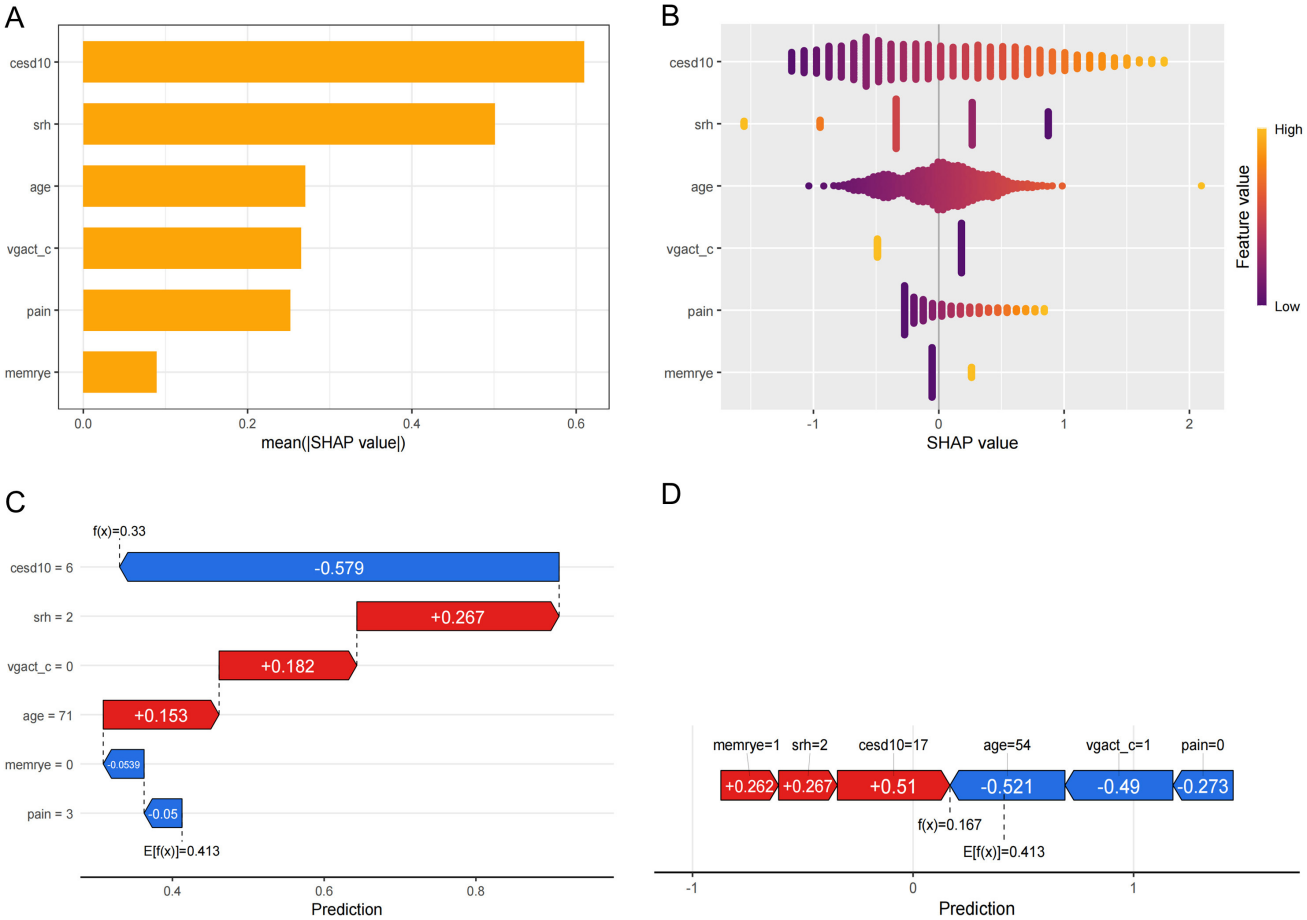
**(A)** Global importance plot; **(B)** Swarm plot; **(C)** Waterfall plots; **(D)** Force plots.

SHAP values were calculated for six key variables in the model. The global importance plot and swarm plot (Figures 5A, B) demonstrated the predictive importance of these variables across the dataset. To further understand their impact at the individual level, waterfall and force plots (Figures 5C, D) were used to visualize the contribution of each variable to the model's predictions in selected samples, highlighting the practical significance of these variables in specific cases.

## 4 Discussion

The medical community has made significant progress in ensuring timely and effective stroke treatment during the acute phase. However, the high disability rate following a stroke remains a critical concern (12). Despite initial treatment, many stroke survivors continue to face challenges with ADL once the acute phase has passed (13). In our analysis of stroke survivors in CHARLS Wave 5, we found that 57.3% of participants exhibited ADL dysfunction. Compared to those with normal ADL function, stroke survivors with ADL dysfunction were generally older, reported shorter sleep durations, experienced higher levels of depressive symptoms, and suffered from poorer physical health. They were also more likely to have multiple chronic conditions, experience bodily pain, and engage in unhealthy habits such as smoking and alcohol consumption. Furthermore, these survivors had lower participation in social activities and physical exercise.

Older stroke survivors are particularly vulnerable to ADL dysfunction (14). Age-related declines in physical, cognitive, and sensory functions can impair one's ability to perform daily tasks independently (15). Research has shown that SRH is closely associated with ADL functioning, with those reporting poorer SRH (e.g., “fair” or “poor”) more likely to experience ADL impairments (16–19). Chronic pain, whether acute or long-term, also plays a significant role in ADL dysfunction, as it can severely hinder daily activities (20, 21). Additionally, chronic pain may exacerbate psychological distress, including depression and anxiety, which can further impair ADL (22). Depression itself, a common mental health issue, contributes to reduced functional capacity and increases the risk of ADL dysfunction (23). Moreover, memory disorders, such as Alzheimer's disease or Parkinson's disease, typically cause gradual cognitive decline, further impairing ADL (24, 25). Conversely, engaging in heavy physical activity has been shown to reduce the likelihood of chronic conditions and can enhance cognitive functioning, which may help prevent age-related cognitive decline (26–29). Therefore, regular physical exercise appears to be a beneficial strategy to improve ADL in stroke survivors. This indicates that heavy physical activities have potential benefits for ADL.

Based on our analysis, we identified six characteristic variables that are commonly observed in stroke survivors. These include age, SRH, heavy physical activity, depression, pain, and memory disorders. Among these variables, age, memory disorders, pain, and depression are known to exacerbate ADL dysfunction, making them significant risk factors for its development. Conversely, ADL dysfunction itself can also contribute to the onset or worsening of limb joint pain and depressive mood in stroke patients, thereby creating a cycle of increasing ADL dysfunction (30). While most stroke survivors experience some degree of ADL dysfunction after the stroke, the severity and progression of this dysfunction can vary (31). For some individuals, improper self-management or the lack of targeted rehabilitation can trigger or intensify ADL dysfunction (32). To address this, the model we developed is designed to identify, at an early stage, the factors closely associated with the onset of ADL dysfunction in this group. Early intervention in these factors is crucial. For example, while direct intervention on age and self-rated health may be challenging, strengthening physical training and improving the management of pain and depression could help prevent or delay the progression of ADL dysfunction. These interventions may also lead to improvements in ADL function, ultimately enhancing the prognosis and quality of life for stroke survivors.

However, our study has several limitations. The CHARLS dataset lacks detailed information on important predictors such as walking pace, grip strength, waist circumference, body mass index (BMI), and certain biochemical markers, which were not captured in Wave 5. Additionally, data on stroke-specific factors such as lesion type, location, size, onset time, and treatment methods are not available in the CHARLS database. Moreover, as the data is specific to our country, the external validity of our findings may be limited, and the models may not be fully applicable to populations in other countries. Internal validation methods were used in this study, but further validation in diverse populations is needed to enhance the generalizability of our predictive models.

## 5 Conclusions

In summary, we developed and validated a prediction model for ADL dysfunction in stroke survivors. The model includes crucial factors like age, SRH, heavy physical activity, depression, pain, and memory disorders. It provides insights into the group's characteristics. Although the study has limitations, the model can guide personalized medical strategies. By implementing these, we can potentially enhance stroke survivors' ADL ability and, consequently, improve their quality of life.

## Data Availability

Publicly available datasets were analyzed in this study. This data can be found here: Utilizing publicly accessible data from the CHARLS, which can be retrieved from the official website at http://CHARLS.pku.edu.cn.
