# Supplementary material for: Development and validation of a risk prediction model for activities of daily living dysfunction in stroke survivors
Source: Front Neurol. 2025 May 30;16:1529724. doi: 10.3389/fneur.2025.1529724 (PMC12164166; doi:10.3389/fneur.2025.1529724)
Supplement: Supplementary file 6 [file Data_Sheet_6.DOC]

library(corrplot)

library(glmnet)

library(caret)

library(CBCgrps)

library(nortest)

library(tidyverse)

library(ggpubr)

library(rms)

library(pROC)

library(ggplot2)

library(mice)

tempdata<-mice(originaldata,m=5,maxit=20,meth='pmm',seed = 500)

tempdata$meth

library(lattice)

densityplot(tempdata)

stripplot(tempdata,pch=20,cex=1.2)

modelfit1<- with(tempdata, glm(diagnosis~gender+marry+rural+hibpe+diabe+

cancre+lunge+hearte+psyche+arthre+

dyslipe+livere+kidneye+digeste+asthmae+memrye+

vgact_c+mdact_c+ltact_c+drinkl+smokev+srh+

family_size+hchild+fcamt+tcamt

+cesd10+satlife+pain+sleep+act+hip+age+edu

+totmet+memeory,

binomial()))

modelfit1

summary(modelfit1,type="glance")

pooled<-pool(modelfit1)

summary(pooled)

data<-complete(tempdata,5)

write.csv(data,"data.csv")

#####table1####

install.packages("scitb")

library(scitb)

library(stringi)

data$diagnosis<- as.factor(data$diagnosis)

allVars<-c("gender", "marry" ,"rural", "hip", "hibpe", "diabe", "cancre",

"lunge", "hearte", "psyche", "arthre", "dyslipe", "livere",

"kidneye",

"digeste","asthmae" ,"memrye", "vgact_c", "mdact_c", "ltact_c",

"drinkl", "smokev","family_size", "hchild", "fcamt", "tcamt",

"cesd10", "satlife", "pain","sleep", "act", "age", "edu",

"totmet","memeory", "srh")###敲上所有变量###

fvars<-c("gender", "marry" ,"rural", "hip", "hibpe", "diabe", "cancre",

"lunge", "hearte", "psyche", "arthre", "dyslipe", "livere", "kidneye"

,"digeste","asthmae" ,"memrye", "vgact_c", "mdact_c", "ltact_c",

"drinkl", "smokev")###敲上所有分类变量###

strata<-"diagnosis"###结局变量###

out<-scitb1(vars=allVars,fvars=fvars,strata=strata,data=data)

setwd("E:/彩图")###输出表格路径###

install.packages("flextable")

install.packages("officer")

library(xtable)

library(flextable)

library(officer)

tb1<-as_flextable(xtable(out))

doc=read_docx()

doc = body_add_flextable(doc,tb1)

print(doc,"./tb1.docx")

###lasso####

#minmax标准化转换

min_max_scale =

function(x){(x-min(x))/(max(x)-min(x))}

data2 = data%>%

mutate(diagnosis = as.factor(diagnosis),

gender=as.factor(gender),

marry=as.factor(marry),

rural=as.factor(rural),

cancre=as.factor(cancre),

hibpe=as.factor(hibpe),

diabe=as.factor(diabe),

lunge=as.factor(lunge),

hearte=as.factor(hearte),

psyche=as.factor(psyche),

arthre=as.factor(arthre),

dyslipe=as.factor(dyslipe),

livere=as.factor(livere),

kidneye=as.factor(kidneye),

digeste=as.factor(digeste),

asthmae=as.factor(asthmae),

memrye=as.factor(memrye),

vgact_c=as.factor(vgact_c),

mdact_c=as.factor(mdact_c),

ltact_c=as.factor(ltact_c),

drinkl=as.factor(drinkl),

smokev=as.factor(smokev),

hip=as.factor(hip),,)%>% ###把所有分类变量变成因子变量

mutate_if(.predicate = is.numeric,

.funs = min_max_scale)%>%

as.data.frame()

#z转化矩阵

set.seed(123) #random number generator

x <- data.matrix(data2[, -1])

y <- data2[, 1]

y<-as.numeric(unlist(y))

lasso <- glmnet(x, y, family = "binomial",nlambda = 1000, alpha = 1)

print(lasso)

plot(lasso, xvar = "lambda", label = TRUE)

#交叉验证

lasso.cv = cv.glmnet(x, y,alpha = 1,nfolds =20,family="binomial")

plot(lasso.cv)

lasso.cv$lambda.min #minimum

lasso.cv$lambda.1se #one standard error away

coef(lasso.cv, s = "lambda.1se") ###非0系数即为危险因素###

###logistic####

##数据集划分

set.seed(1)

train_id = sample(1:nrow(data),0.6*nrow(data))

train=data[train_id,]

test=data[-train_id,]

write.csv(train,file="train.csv")

write.csv(test,file="test.csv")

###logistics和列线图###

mydata<-train

attach(mydata)

dd<-datadist(mydata)

options(datadist='dd')

fit<-lrm(diagnosis ~ gender

+vgact_c

+mdact_c

+memrye

+hip

+cesd10

+pain

+act

+age

+srh

+memeory,

data = mydata, x = T, y = T)

fit ###多因素回归取p值小于0.05###

install.packages("devtools")

install.packages(c('fields', 'R2HTML', 'kernlab', 'ROCR'))

library(VRPM)

library(survival)

setwd("E:/彩图")

mydata<-train

fit <-glm(diagnosis ~

vgact_c

+memrye

+cesd10

+pain

+age

+srh,data=mydata,

family ="binomial")

colplot(fit)

colplot(fit,coloroptions = 1)

###ROC####

gd<-predict(fit, newdata = train,

se.fit = FALSE, dispersion = NULL, terms = NULL,

na.action = na.pass)

gd2<-predict(fit, newdata = test,

se.fit = FALSE, dispersion = NULL, terms = NULL,

na.action = na.pass)

library(pROC)

library(ggplot2)

##训练集的ROC#####

roc.list1 <- roc(train$diagnosis, gd)

roc.list1

auc <- ci.auc(roc.list1)

auc

best1=coords(roc.list1, "best",best.method = c("youden"),

ret=c("threshold","sensitivity", "specificity"))

best1

g.list1 <- ggroc(roc.list1, alpha = 1 ,size = 1,

legacy.axes = TRUE,color="#7ec5f4")

p <- g.list1+theme_replace() + #设置主题

annotate(geom = "segment", x = 0, y = 0, xend =1, yend = 1,linetype=2)+#设置对角线

###加上AUC和95%CI

annotate("text", x = 0.75 , y = 0.25,

label = paste("AUC=",round(auc[2],3)),

colour="#5d6174",size=7)+#展示AUC

annotate("text", x = 0.75 , y = 0.18,

label = paste("95%CI:",round(auc[1],3),"-",round(auc[3],3)), colour="#5d6174",size=7)+

###加上最佳截断值的文字

annotate("text", x = 1-best1$specificity , y = best1$sensitivity+0.1,

label = paste("Cutoff=",best1$threshold),colour="red2",size=7)+

###加上最佳截断值的点

geom_point(x=1-best1$specificity,y=best1$sensitivity,color="red3",size=3)+

#标出截断值 ###坐标轴的相关参数设置

theme(axis.text = element_text (size = 20))+#调整坐标轴字体大小

theme(axis.title.x=element_text(vjust=2, size=20,face = "plain"))+#调整xlab字体大小

theme(axis.title.y=element_text(vjust=2, size=20,face = "plain"))+#调整ylab字体大小

theme(axis.line = element_line(color = "black",linewidth = 1))#调整坐标轴样式

p

###导出

ggsave("ROC.pdf", plot = p, width = 6, height = 6)

ggsave("ROC.png", plot = p, width = 6, height = 6,dpi = 300)

##验证集的ROC#####

roc.list2 <- roc(test$diagnosis, gd2)

roc.list2

auc <- ci.auc(roc.list2)

auc

best2=coords(roc.list2, "best",best.method = c("youden"),

ret=c("threshold","sensitivity", "specificity"))

best2

g.list2 <- ggroc(roc.list2, alpha = 1 ,size = 1,

legacy.axes = TRUE,color="#7ec5f4")

p <- g.list2+theme_replace() + #设置主题

annotate(geom = "segment", x = 0, y = 0, xend =1, yend = 1,linetype=2)+#设置对角线

###加上AUC和95%CI

annotate("text", x = 0.75 , y = 0.25,

label = paste("AUC=",round(auc[2],3)),

colour="#5d6174",size=7)+#展示AUC

annotate("text", x = 0.75 , y = 0.18,

label = paste("95%CI:",round(auc[1],3),"-",round(auc[3],3)), colour="#5d6174",size=7)+

###加上最佳截断值的文字

annotate("text", x = 1-best2$specificity , y = best2$sensitivity+0.1,

label = paste("Cutoff=",best2$threshold),colour="red2",size=7)+

###加上最佳截断值的点

geom_point(x=1-best2$specificity,y=best2$sensitivity,color="red3",size=3)+

#标出截断值 ###坐标轴的相关参数设置

theme(axis.text = element_text (size = 20))+#调整坐标轴字体大小

theme(axis.title.x=element_text(vjust=2, size=20,face = "plain"))+#调整xlab字体大小

theme(axis.title.y=element_text(vjust=2, size=20,face = "plain"))+#调整ylab字体大小

theme(axis.line = element_line(color = "black",linewidth = 1))#调整坐标轴样式

p

###导出

ggsave("ROC.pdf", plot = p, width = 6, height = 6)

ggsave("ROC.png", plot = p, width = 6, height = 6,dpi = 300)

#####校准曲线######

library(rms)

fit1<-lrm(diagnosis ~

vgact_c

+memrye

+cesd10

+pain

+age

+srh,

x=TRUE,y=TRUE,data=train)

cali<-calibrate(fit1,B=400)

plot(cali)#####训练集校准曲线######

fit2<-lrm(diagnosis~

vgact_c

+memrye

+cesd10

+pain

+age

+srh,

x=TRUE,y=TRUE,data=test)

cali<-calibrate(fit2,B=400)

plot(cali)#####测试集校准曲线######

#####决策曲线#####

install.packages("ggscidca")

library(ggscidca)

library(survival)

library(reshape2)

library(ggplot2)

model1<-glm(diagnosis ~

vgact_c

+memrye

+cesd10

+pain

+age

+srh,family

=binomial(link="logit"),data=train)

model2<-glm(diagnosis ~

vgact_c

+memrye

+cesd10

+pain

+age

+srh,family

=binomial(link="logit"),data=test)

scidca(model1,newdata = train,modelnames = "train")#####训练集DCA#####

scidca(model2,newdata = test,modelnames = "test")####测试集DCA####
